# Neck Circumference in Overweight/Obese Subjects who Visited the Binjai Supermall in Indonesia

**DOI:** 10.3889/oamjms.2016.072

**Published:** 2016-07-16

**Authors:** Dharma Lindarto, Shierly, Santi Syafril

**Affiliations:** *Department of Internal Medicine, Faculty of Medicine, North Sumatra University - H. Adam Malik Hospital, Jalan Bunga Lau No 17, Medan 20136, Indonesia*

**Keywords:** obesity, neck circumference, cutoff point, overweight, BMI

## Abstract

**BACKGROUND:**

Neck circumference (NC) is a simple screening measure for identifying overweight and obesity, it reflects upper-body fat distribution and central obesity.

**AIM:**

To determine whether a single measure of NC might be used to identify overweight/obesity.

**MATERIAL AND METHODS:**

An observational, analytical, cross-sectional study was done. The subjects consisted of all consecutive subjects who visited Binjai Supermall (North Sumatera Province, Indonesia) between 23rd and 29th September 2015 and agreed to participate in the study. NC, weight, height, body mass index (BMI), and waist circumference (WC) were measured. Overweight and obesity were defined as BMIs of 23.0–24.9 and ≥ 25 kg/m^2^, respectively.

**RESULTS:**

In total, 1554 subjects participated. Of these, 1238 (79.7%) were overweight/obese. NC correlated significantly with weight, height, BMI, and WC. Receiver operating characteristic (ROC) analysis showed that for all men and women, the area under the curve of overweight/obesity for NC was 0.83 and 0.79, respectively. The best NC cutoff points for males and females that indicated overweight/obesity were ≥ 37 cm (sensitivity, 78.3% and specificity, 75.5%) and ≥ 33.5 cm (sensitivity, 76.6% and specificity, 66.7%), respectively.

**CONCLUSION:**

The NC cutoffs that were identified may be useful for screening for overweight/obesity and related co-morbidities.

## Introduction

Obesity is defined as an excessively high amount of body fat or adipose tissue in relation to lean body mass [1]. Current estimates; in the US, are that 69% of adults are either overweight or obese, with approximately 35% being obese [2]. Obesity raises the risk of hypertension, dyslipidemia, type 2 diabetes mellitus (T2DM), coronary heart disease, stroke, gallbladder disease, osteoarthritis, sleep apnea, respiratory problems, and some cancers [3]. In developing countries, people with a high socioeconomic status are most likely to be obese. This may be due to their occupation, education level, physical activity, and tendency to smoke [4]. In 2013, the prevalence of obesity in North Sumatra Province, Indonesia was about 27%. This is markedly greater than the national prevalence of obesity (20%) [5].

When assessing obesity, various techniques are used. These include measuring BMI, the waist circumference (WC), waist/hip ratio, mid-upper arm circumference, subscapular/triceps ratio, and neck circumference (NC) [6]. NC is a particularly simple and rapidly obtained anthropometric measurement that can be used to screen for overweight/obese people because it reflects upper-body fat distribution and central obesity [7, 8]. The Framingham Heart Study showed that NC is an index of central obesity as it associates independently with visceral adiposity and body mass index (BMI). Moreover, NC associates independently with cardiometabolic risk factors (*e.g*., systolic and diastolic blood pressure) even after adjusting for visceral adiposity or BMI and WC [9]. Similarly, NC with metabolic syndrome correlated better among females than males. The cross-sectional study was conducted in a tertiary care hospital in South India, showed that men with NC >37 cm and women with NC >34 cm are more prone to cardiometabolic syndrome and require additional evaluation [10]. A study of 3182 diabetic Chinese patients showed that NC correlated positively with BMI, WC, and metabolic syndrome [11]. However, the usefulness of the NC measurement in diverse healthy and clinical populations in Indonesia has not yet been reported.

The aim of this study was to determine whether a single measure of NC might be used to identify overweight/obese and to define NC cutoff levels for overweight/obesity for men and women according to existing BMI as standards.

## Material and Methods

An observational, analytical, cross-sectional study was done. The study was approved by local of the Research and Ethics Committee and was conducted according to the principles of Helsinki and its revisions. All participants provided written informed consent to participate in the study before enrollment in the study.

### Subjects

The subjects were all consecutive participants who visited Binjai Supermall (North Sumatera Province, Indonesia) between 23rd and 29th September 2015 and agreed to participate in the study after its objectives and methods were explained. The subjects were recruited by a team composed of three trained interviewers who stood near the main entrance of the Supermall next to a 3×4 m billboard bearing the words “Survey Obesity” (“Survey of Obesity”). The interviewers explained the purpose of the study to passers-by. If people agreed to participate, they were led to a secluded room staffed by three medical professionals who took the subject’s medical history and determined their eligibility to participate. Subjects were excluded if they were pregnant or breastfeeding, had a history of neck disease (*e.g*., thyroid disorders, neck surgery, or neck malignancy), had a history of lung diseases such as chronic obstructive pulmonary disease, asthma, or pulmonary fibrosis, or had any anatomical disorder. Written informed consent was obtained. Thereafter, the anthropometric measurements (height, weight, BMI, WC, and NC) were obtained.

### Anthropometric Evaluation

The anthropometric measurements were obtained using standard techniques. WC was measured at the midpoint between the lowest costal margin and the iliac crest at the end of normal expiration [3]. For women, NC was measured in the middle of the neck, namely, between the mid cervical spine and the mid anterior neck. For men, NC was measured just below the laryngeal prominence (Adam’s apple) [12]. All measurements were taken in the standing position. Weight was measured with a digital scale while the patient was wearing light clothing and lacked shoes. The digital scale measured weight to the nearest 100 g. Height was measured with a stadiometer while the subject was without shoes.

### Definitions of Anthropometric Measurement Cutoffs

Healthy WC limits are 90 cm for men and 80 cm for women. BMI was calculated by dividing weight (kg) by the square of height (m^2^). Normal weight and underweight were considered to be BMIs of 18.5– 22.9 and < 18.5 kg/m^2^, respectively. Overweight and obesity were considered to BMIs of 23.0 – 24.9 and ≥ 25 kg/m^2^, respectively. These weight cutoffs were specified for the Asia-Pacific population by the Western Pacific Regional Office of the World Health Organisation [13].

### Statistical Analysis

Data are shown as mean ±SD unless otherwise specified. Independent t-test and Pearson’s correlation were the tests of significance done to analyse the quantitative data. The NC cutoffs for overweight/obesity against BMI in males and females were identified by receiver operator characteristic (ROC) analysis. A p-value of less than 0.05 was considered statistically significant. All statistical analysis was performed using SPSS 22.0 software.

## Results

### Characteristics of the Participants

In total, the study sample of 1554 subjects who visited Binjai Supermall. Of these, 1238 (79.7%) were overweight/obese (554 males and 684 females, aged between 25 and 70 years old).

The overweight/obese males and females did not differ significantly in terms of age, but the males were significantly heavier and taller and had larger NCs and WCs. However, the males and females did not differ in terms of BMI (Table 1).

**Table 1:** Anthropometric measurements of the overweight/obese participants in the study population

Characteristics	All Overweight/Obese *Mean* ± SD	Male *Mean* ± SD	Females *Mean* ± SD	P-value
Sex (M/F)	1238	554	684	
Age (yr)	41.6 ± 10.34	41.6 ± 10.72	41.6 ± 10.03	0.965
Weight (kg)	72.4 ± 11.89	78.2 ± 11.89	67.8 ± 9.67	0.000**
Height (cm)	161 ± 9.00	167.6 ± 6.85	155.5 ± 6.65	0.000**
NC (cm)	37.4 ± 3.46	39.1 ± 2.86	36.2 ± 3.32	0.000**
WC (cm)	92.7 ± 10.00	95.9 ± 9.77	90.1 ± 9.42	0.000**
BMI (kg/m^2^)	27.9 ± 3.58	27.7 ± 3.52	28.1 ± 3,63	0.180

Data are expressed in mean ± SD. Abbreviations: NC: neck circumference; BMI: body mass index; WC: waist circumference; * P<0.05,

** P<0.01.

Correlation analyses in the whole cohort (n = 1238), all males, and all females, NC correlated positively and significantly with weight (males: r = 0.621, P = 0.000; females: r = 0.452, P = 0.000), height (males: r = 0.218, P = 0.000; females: r = 0.195, P = 0.000), WC (males: r = 0.650, P = 0.000; females: r = 0.458, P = 0.000), and BMI (males: r = 0.578, P = 0.000; females: r = 0.373, P = 0.000) (Table 2).

**Table 2:** Correlations between neck circumference and other anthropometric variables in the whole cohort and in males and females only

Correlation	Total (n =1238)		Males (n = 554)		Females (n = 684)	
	r	P	r	P	r	P
Weight	0.595	0.000**	0.621	0.000**	0.452	0.000**
Height	0.452	0.000**	0.218	0.000**	0.195	0.000**
WC	0.570	0.000**	0.650	0.000**	0.458	0.000**
BMI	0.357	0.000**	0.578	0.000**	0.373	0.000**

Abbreviations: NC: neck circumference; BMI: body mass index; WC: waist circumference; * P<0.05,

** P<0.01.

**Figure 1:**
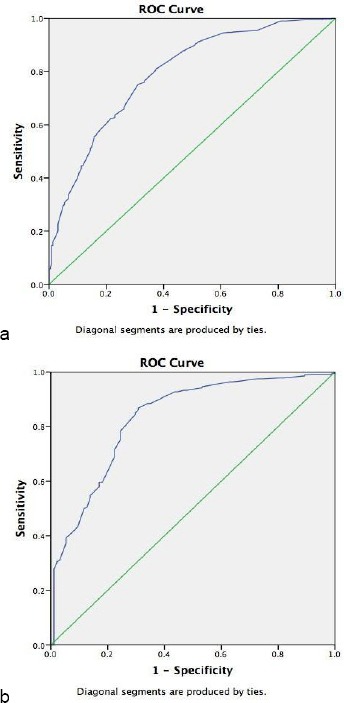
Receiver operating characteristic curves related to neck circumference and BMI in males (a) and females (b)

ROC analysis showed that the area under the curve (AUC) of overweight/obesity for NC and BMI was 0.83; 95% CI 0.78-0.87; P < 0.001 (Figure 1) and 0.79; 95% CI 0.75–0.82; P < 0.001 (Figure 2) for men and women, respectively.

When BMI served as the standard of overweight/obese, the best NC cutoff points for males and females were ≥ 37 cm (sensitivity, 78.3% and specificity, 75.5%), and ≥ 33.5 cm (sensitivity, 76.6% and specificity, 66.7%), respectively (Table 3).

**Table 3:** Neck circumference cutoff levels that indicate overweight/obese males and females, as determined by Receiver Operating Characteristic analysis

Cutoff	Males		Cutoff	Females	
	Sensitivity (%)	Specificity (%)		Sensitivity (%)	Specificity (%)
36.45	88.4	66.0	33.05	81.3	62.2
36.55	86.8	69.1	33.15	81.1	62.6
36.65	86.3	69.1	33.25	80.1	63.1
36.80	85.0	70.2	33.35	79.6	63.5
36.95	84.5	70.2	33.45	79.2	64.0
**37.05**	**78.3**	**75.5**	**33.55**	**76.6**	**66.7**
37.15	78.0	75.5	33.65	76.3	66.7
37.30	76.2	75.5	33.75	76.0	66.7
37.45	75.3	75.5	33.85	75.1	68.9
37.55	71.3	77.7	33.95	74.5	69.4
37.65	70.8	77.7	34.05	67.5	73.4

Comparison of this study with previously conducted studies in Table 4.

**Table 4:** Comparison of the results of this study with previously conducted studies

Parameter	Sex	This Study N = 1238	Aswathappa et al [19] N = 1351	Hingorjo et al [20] N = 155	Ang NS & Raboca JC [21] N = 224
Study Population	Male	554	840	41	116
	Female	684	511	109	108
BMI (kg/m^2^)	Male	27.9 ± 3.58	24.8 ± 4.40	21.69 ± 4.93	27.8 ± 3.68
	Female	27.7 ± 3.52	25.38 ± 4.89	21.04 ± 3.82	25.38 ± 4.89
WC (cm)	Male	95.9 ± 9.77	90.47 ± 12.09	80.67 ± 12.94	95.0 ± 8.73
	Female	90.1 ± 9.42	86.56 ± 12.09	78.17 ± 9.12	86.56 ± 12.09
NC (cm)	Male	39.1 ± 2.86	36.4 ± 5.70	35.56 ± 2.77	37.6 ± 3.81
	Female	36.2 ± 3.32	34.1 ± 5.70	31.52 ± 1.96	34.12 ± 5.70
Cutoff NC (cm)	Male	≥ 37	≥ 36	≥ 35.5	≥ 40
	Female	≥ 33.5	≥ 32	≥ 32	≥ 33.8
Determining the overweight/ obese		BMI ≥ 23 kg/m^2^	BMI ≥ 23 kg/m^2^	BMI ≥ 23kg/m^2^	WC ≥ 90cm (males)

Abbreviations: NC: neck circumference; BMI: body mass index; WC: waist circumference.

## Discussion

Obesity is now reaching pandemic proportions across much of the world and will impose an unprecedented health, financial, and social burden on the general public unless effective actions are taken to reverse the trend [14]. In 1956, Vague was the first researcher to find that the thickness of a neck skinfold, as measured by callipers, is a marker of upper-body fat distribution [15]. Thereafter, it was shown that NC can serve as an anthropometric marker to screen for central obesity and upper-body subcutaneous fat and their related co-morbidities [14]. Indeed, NC seems superior to other central obesity indicators in terms of predicting related co-morbidities: after adjustment for BMI and WC, NC was found to be the only risk factor for T2DM [14]. Similarly, Vallianou *et al*. showed that NC associated independently with high-density lipoprotein cholesterol, glucose, triglyceride, and uric acid levels, even after adjusting for BMI and WC. This indicates that NC may also be useful for screening atherogenic dyslipidemia [16]. The usefulness of this variable is heightened by the fact that it is easy to measure and thus can be used for self-monitoring by lay people.

The present study showed that NC correlated strongly and positively with weight, height, BMI, and WC in both male and female subjects. Several studies have also examined the association between conventional anthropometric measures of obesity and NC [6, 11, 17, 18]. In particular, Papandreou *et al* reported that NC was found to be independently associated with obesity levels in Emirati college students [19], Onat *et al*. reported that NC correlated strongly with BMI, WC, homeostatic model-assessed insulin resistance, and blood pressure [17]. Similarly, Yang *et al*.showed that, in Chinese subjects with T2DM, NC correlated positively with BMI, WC, and metabolic syndrome [11].

The present study showed that the NC cutoff points that best-indicated overweight/obese males and females were ≥ 37 cm (sensitivity, 78.3% and specificity, 75.5%) and ≥ 33.5 cm (sensitivity, 76.6% and specificity, 66.7%), respectively. These findings are consistent with similar studies performed in India [20] and Pakistan [21]. However, our cutoff points were lower than those generated by studies in countries such as the Philippines [22] and Iran [23]. This may be due to the fact that the present study used Asian BMI cutoff values to identify the NC cutoff points: in Asians, the BMI cutoff that indicates overweight/obesity is > 23.0 kg/m^2^, whereas, in Caucasians, it is > 25 kg/m^2^.

The study has several limitations that should be addressed in future research. In particular, the subjects who were included in this study were not evaluated in terms of their metabolic profile or thyroid function. Thus, whether the NC cutoffs we identified could be used to predict the risk of metabolic syndrome is not yet clear.

In conclusion, this study indicated that 79.7% of the visitors to Binjai Supermall during a 7 days period in 2015 were overweight/obese, as indicated by their BMI. NC correlated strongly with weight, height, BMI, and WC. When BMI cutoffs for the Asia-Pacific population were used in ROC analysis, the best NC cutoffs for indicating overweight/obesity in males and females were ≥ 37 cm (sensitivity, 78.3% and specificity, 75.5%) and ≥ 33.5 cm (sensitivity, 76.6% and specificity, 66.7%), respectively. These values may be useful because NC predicts overweight and obesity and related co-morbidities and can be used as an initial screening tool for the purpose. This usefulness is enhanced by the fact that it is a straightforward and inexpensive test that can be performed in any office with a tape measure.
